# Non-Pharmacological Therapeutic Options for Liver Metastases in Advanced Neuroendocrine Tumors

**DOI:** 10.3390/jcm8111907

**Published:** 2019-11-07

**Authors:** Solène Dermine, Lola-Jade Palmieri, Julie Lavolé, Amélie Barré, Antony Dohan, Einas Abou Ali, Anne-Ségolène Cottereau, Sébastien Gaujoux, Catherine Brezault, Stanislas Chaussade, Romain Coriat

**Affiliations:** 1 Gastroenterology and Digestive Oncology, Cochin Hospital, Assistance Publique-Hôpitaux de Paris, 75014 Paris, France; lolajade.palmieri@aphp.fr (L.-J.P.); julie.lavole88@gmail.com (J.L.); amelie.barre@aphp.fr (A.B.); einas.abouali@aphp.fr (E.A.A.); catherine.brezault@aphp.fr (C.B.); stanislas.chaussade@aphp.fr (S.C.); 2 Department of Gastroenterology, Cochin Teaching Hospital, Université de Paris, 75014 Paris, France; anthony.dohan@aphp.fr (A.D.); annesegolene.cottereau@aphp.fr (A.-S.C.); sebastien.gaujoux@aphp.fr (S.G.); 3 Department of Radiology, Cochin Hospital, Assistance Publique-Hôpitaux de Paris, 75014 Paris, France; 4 Department of Nuclear Medicine, Cochin Hospital, Assistance Publique-Hôpitaux de Paris, 75014 Paris, France; 5 Digestive Surgery Unit, Cochin Hospital, Assistance Publique-Hôpitaux de Paris, 75014 Paris, France

**Keywords:** neuroendocrine tumors, liver metastasis, surgery, radiofrequency ablation, trans-arterial embolization, trans-arterial chemoembolization, trans-arterial radioembolization

## Abstract

The incidence of liver metastasis in digestive neuroendocrine tumors is high. Their presence appears as an important prognostic factor in terms of quality of life and survival. These tumors may be symptomatic because of the tumor burden itself and/or the hormonal hyper-secretion induced by the tumor. Surgery is the treatment of choice for resectable tumors and metastasis. Nevertheless, surgery is only possible in a small number of cases. The management of non-resectable liver metastasis is a challenge. The literature is rich but consists predominantly in small retrospective series with a low level of proof. Thus, the choice of one technique over another could be difficult. Local ablative techniques (radiofrequency) or trans-catheter intra-arterial liver-directed treatments (hepatic artery embolization, chemo-embolization, and radio-embolization) are frequently considered for liver metastasis. In the present review, we focus on these different therapeutic approaches in advanced neuroendocrine tumors, results (clinical and radiological), and overall efficacy, and summarize recommendations to help physicians in their clinical practice.

Neuroendocrine tumors (NETs) are rare and represent 1% of tumors, but their incidence, 3.56/100.000 people per year, is constantly increasing due to better detection of small tumors [1]. They are derived from embryonic neural crest tissue and can be functioning (by secretion of one or more biologically active peptides) or nonfunctioning. Most NETs are from the digestive origin (58%), with a variable frequency (small intestine, pancreas, colorectal, appendix, pancreas, stomach) [2,3]. Gastrointestinal functioning tumors are insulinomas, gastrinomas, vasoactive intestinal peptide tumor (VIPomas), glucagonomas and somatostatinomas, and NETs with carcinoid syndrome (due to serotonin or other peptide secretion in the setting of liver metastases) and can be diagnosed by their hormonal syndrome. The WHO classification 2017 defined four different prognosis groups of tumors, according to cell differentiation and the proliferation index (Ki-67 index) [4]. Grade 1 (Ki-67 < 3%) and 2 tumors (Ki-67, 3%–20%) are the majority and well differentiated. Well-differentiated grade 3 (Ki-67 > 20%) tumors are rare [5]. Tumors with poor differentiation and high Ki-67 (Ki-67 > 20%) are called neuroendocrine carcinoma in front of their pejorative prognosis. 

In total 40% to 50% of NETs patients present with metastasis at the initial diagnosis. Gastrointestinal NETs are predisposed to metastasize in the liver and/or lymph nodes [6]. Liver metastases (LM) impair the patient’s quality of life and are associated with poor prognosis [7,8], with a median overall survival of 5 to 57 months [9]. Morphological imaging (computed tomography scan, magnetic resonance imaging) shows the presence of hypervascular lesions with hepatic arterial phases in NETs. Meanwhile, NETs may appear as hypovascular or cystic lesions [10]. Furthermore, functional imaging with position emission tomography (PET) using radiolabeled somatostatine analogues combined with computed tomography can be performed to better characterize these lesions: Somatostatin receptor scintigraphy or (68)Ga-[tetraxetan-D-Phe1, Tyr3]-octreotide PET (^68^Ga-DOTATOC) for pancreatic NETs, 18F-fluoro-dihydroxyphenylalanine PET (^18^F-DOPA PET) for small-bowel NETs, and (18)F-fluorodeoxyglucose (FDG) PET (^18^FDG PET) for neuroendocrine carcinoma. Functional imaging represents the gold standard approach to characterize these lesions because it may detect lesions that morphologic imaging or somatostatin-receptor scintigraphy cannot [10,11] (Figure 1). The role of imaging in the management of patients with LM is important as it assesses the number and distribution of metastases, and their location according to the hepatic vessels [12]. Furthermore, functional imaging can detect extra-hepatic disease with a good sensitivity and specificity, which may change clinical strategy. This is especially relevant in the case of liver transplantation strategy [13].

Due to the rarity of the disease, the number of prospective randomized trials is limited, and most recommendations are based on retrospective studies with a low evidence-based medicine. The NETs population is heterogeneous because of the heterogeneity of this disease and the difficulty of defining cohorts of patients with the same pathological classification of disease. The majority of studies include a small number of patients. There are no studies comparing the different therapies to each other in NETs LM.

Treatment of patients with LM is aimed at local tumor control and symptom relief. There are several therapeutic options and treatment is complex. The management of NETs patients with LM can include surgical treatment, loco-regional medical therapies, nuclear medicine interventions, and/or pharmacological treatment. This broad range of possible therapies necessitates recommendations for the optimal treatment of these patients [8]. Our review focuses on intestinal and pancreatic NETs. The objective was to describe the role of liver-directed treatments in patients with LM attributable to NETs.

## 1. Therapeutic Options for LM

### 1.1. Surgery

#### 1.1.1. Techniques

Two kinds of surgery can be used in the case of LM. Curative surgery is performed when complete resection is possible. Debulking or cytoreductive surgery or partial (R2) surgical resection are performed when the majority of the tumor (at least 90% of the hepatic tumor mass) is removed to control the symptoms of the disease, most commonly in functional tumors. This surgery is indicated in patients with symptoms that are not controllable with medical or hormonal treatments.

#### 1.1.2. Results

Complete resection of LM is the sole curative treatment and is possible in only 10% to 25% of patients [7,8]. Bilobar metastases may be treated with two-step resections. In this case, preoperative portal vein embolization can be performed to induce hypertrophy of the left liver lobe [14]. Five-year survival rates of 46% to 86% have been reported in the case of surgery. There is a high rate of disease recurrence within 3 to 5 years even if surgery is performed with curative intent, but 5-year survival rates approach 85% [15]. No randomized trials have compared liver resection to other treatments in NETs LM, but retrospective studies highlight the advantage of surgical treatment. Debulking was performed to control symptoms and improve the quality of life. Recurrence of symptoms occurs in the first 5 years [16]. Debulking surgery is associated with improved survival in some retrospective studies [17,18]. However, comparative trials to systemic therapy are lacking, so the superiority of debulking surgery in the case of non-functional tumors cannot be affirmed. Surgery for a limited number of liver metastases preferably localized to one lobe is recommended by the ENETS (European Neuroendocrine Tumor Society) and NANETS (North American Neuroendocrine Tumor Society) guidelines to improve quality of life and survival. Regarding cytoreductive surgery, guidelines conclude that surgical resection should be attempted if the disease is not progressive over a 6-month period in patients suffering from symptoms related to tumor burden and when at least 90% of the visible tumor can be removed, or in uncontrolled functional tumors [7,19,20].

#### 1.1.3. Complications

Morbidity and mortality of surgery are acceptable and comparable to other liver resections. Sarmiento et al. identified a post-operative mortality of 0%, complications rate of 18%, and a 5-year survival of 71% [15]. 

### 1.2. Liver Transplantation

#### 1.2.1. Techniques

Liver transplantation consists of a total hepatectomy and orthotopic liver transplantation at a single time-point. This technique is rarely used and represents only 0.2% to 0.3% of all liver transplants recorded in US/European liver transplant registries [21,22]. Liver transplantation has been proposed for two reasons: The lower aggressivity of NETs and the low percentage of patients with free-margins (R0) liver resections. 

#### 1.2.2. Results

Liver transplantation represents a potentially curative treatment [23,24,25,26]. Recent data have suggested that liver transplantation may represent the most efficient approach in terms of overall and disease-free survival, but patients’ and primary tumors’ characteristics are poorly described. A recent systematic review of retrospective case series calculated a median overall survival at 1, 3, and 5 years of 89%, 69%, and 63%, respectively. Recurrence after liver transplantation ranged between 31.3% and 56.8% [24]. Gedaly et al. calculated 1-, 3-, and 5-year overall survival rates of 81%, 65%, and 49%, respectively. Recurrence information was available for 83 patients, and 1-, 3-, and 5-year disease-free survival rates were 77%, 50%, and 32%, respectively [21]. In Le Treut et al.’s study, the median overall survival post-liver transplantation was 67 months, with 1-, 3-, and 5-year overall survival rates of 81%, 65%, and 52%, respectively. Disease-free survival rates at the same intervals were 65%, 40%, and 30%, respectively [22]. These larger studies are limited by the heterogeneity of the included patients. The selection criteria used are poorly documented. A preselection of patients for liver transplantation may increase 5-year survival rates [23,24,25,26]. No study addressed the quality of life after liver transplantation. Due to the small number of liver transplantations in this indication, the role of liver transplantation was controversial and cannot be recommended by ENETS and NANETS guidelines [7,19,20]. Furthermore, the indications are limited because of the difficulty in the allocation of organs to oncological patients and the lack of donors. 

#### 1.2.3. Complications

The 3-month post-operative mortality was estimated to be up to 10% [22]. Major complications in the early post-transplant period include biliary anastomotic leakage, septicemia, liver failure, hematoma, and hepatic or artery thrombosis. Also, in the late follow-up period, a biliary anastomotic stricture may occur within 6 months after transplant.

### 1.3. Ablative Techniques (Radiofrequency Ablation and Other Ablative Techniques)

#### Techniques

Radiofrequency ablation is a thermal ablative technique based on the cytotoxic effects of high temperature locally administrated in the liver. A high-frequency current is transmitted to the liver through electrode needles, and the ionic vibrations generated by the high frequency induce coagulation necrosis. Radiofrequency ablation can be performed percutaneously under imaging guidance or intraoperatively. Computed tomography scan or magnetic resonance imaging is used to assess complete tumor necrosis [27]. Radiofrequency ablation series document a 5-year overall survival of 53% [8]. Furthermore, radiofrequency ablation is often associated with surgery [28,29]. Elias et al. reported an overall survival rate of 84% at 3 years [28]. Akyildiz et al. reported a median disease-free survival of 1.3 years and overall survival of 6 years after radiofrequency ablation. Local liver recurrence was observed in 7.9%, but new tumors are reported in up to 63%. Improvement in symptom control was reported, with a rate of 97% in symptom response [29]. Major complications observed after radiofrequency ablation include portal vein thrombosis, hemoperitoneum, colonic perforation, liver abscess, and tumor seeding. Minor complications include pain, segmental biliary dilatation, pleural effusions, and sub-capsular hematoma [29,30]. Indeed, radiofrequency ablation may be contraindicated for lesions around vital structures or the liver surface. Radiofrequency ablation in the case of bilio-enteric anastomosis increases the risk of liver abscess formation (40% vs. 0.4%) [30]. Despite this complication, radiofrequency ablation appears to be a safe technique and a well-tolerated procedure with low morbidity and mortality rates. Akyildiz et al. reported a perioperative morbidity of 6% and a 30-day mortality of 1% [29]. 

Other ablative techniques are mainly represented by microwaves ablation, cryotherapy, and percutaneous ethanol injection. Microwave ablation uses electromagnetic devices with high frequencies, similar to radiofrequency ablation. A prospective series reported 11 NETs patients with LM. No local recurrence was noticed. Complications were observed in three patients [31]. Cryotherapy and percutaneous ethanol injection are alternatives in cases in which tumors are close to vital structures or vessels. Cryotherapy is based on necrosis in tumor tissue at low temperatures. Bilchik et al. described 19 patients who received cryotherapy. The reduction in tumor markers reached 90%. Median symptom-free and overall survival were 10 months and more than 49 months, respectively. Coagulopathy post-cryotherapy was found in all patients, treated by an infusion of fresh frozen plasma or platelets. There were no operative or perioperative deaths [32]. Percutaneous ethanol injection induces chemical destruction by an intra-tumor injection of alcohol. This technique is mostly used in hepatocellular carcinoma, and is proposed for LM of NETs by some experts [33]. These techniques have been replaced by radiofrequency ablation for safety reasons. Ablative techniques should be considered as a treatment option, in association or not with surgery, in carefully selected patients according to the NANETS guidelines [19,20].

### 1.4. Trans-Arterial Embolization and Chemoembolization

#### 1.4.1. Techniques

**Figure 2:**
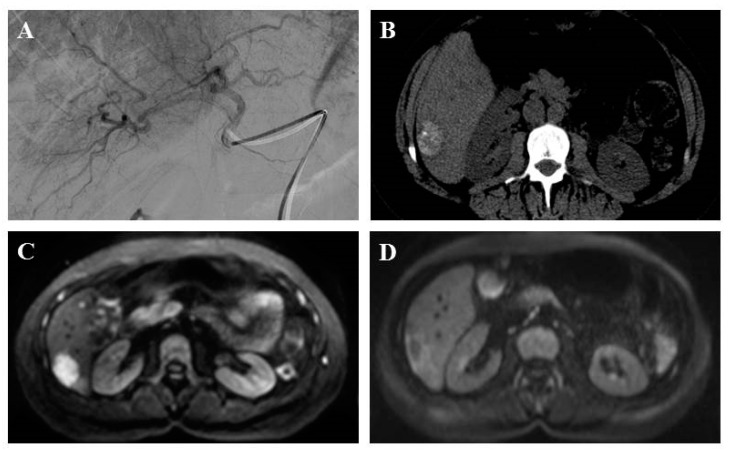
A 51-year-old patient with liver metastasis from pancreatic grade 2 neuroendocrine tumor treated with lipiodol and doxorubicin chemoembolization. Prior to chemoembolization, the arteriography of tumor-feeding branches (2**A**) and diffusion-weighted axial plane magnetic resonance imaging (b = 1000 s/mm^2^ with restriction of diffusion) (2**B**) showed a right liver lesion. Axial computed tomography scan without injection showed a major lipiodol uptake after one month (2**C**). Diffusion-weighted axial plane magnetic resonance imaging (b = 1000 s/mm^2^) showed a complete response at three months (2**D**).

These treatments consist in the intravascular delivery of agents via selective catheter placement with imaging guidance, because LM are highly vascularized (and almost exclusively) by the hepatic artery while normal parenchyma is vascularized by the portal vein [34]. Trans-arterial embolization (TAE) involves the administration of embolic agents like lipiodol, absorbable gelfoam particles, polyvinyl alcohol (PVA) foam, or non-absorbable bland microspheres (Embosphere®), which will stop the arterial blood flow into the hepatic artery and its branches, for a limited time, up to the hypervascular nodules during an angiography (Figure 2). Trans-arterial chemoembolization (TACE) is performed in two steps: An injection of a mixture between a vector (oil, Lipiodol®) and a chemotherapy agent (usually doxorubicin, 50 mg/m^2^, or streptozotocin, 1.5 g/m^2^) or a combination of chemotherapy agents (cisplatin + doxorubicine, mitomycin C + cisplatine + doxorubicin) diluted in conventional trans-arterial chemoembolization saline, then an embolization. Two kinds of effects are expected: A toxic effect by progressive release of high concentrations of the chemotherapy agent in tumor disease and an ischemic effect. Embolization also slows the blood flow, increasing the local chemotherapy concentration and drug retention in the tumor, over 20 times greater than with systemic administration [35,36]. New chemoembolization techniques, such as drug-eluting beads (DEBs, non-absorbable pre-loaded particles), also use both mechanisms with progressive release into the tumor of the highly-concentrated chemotherapy agent and micro-embolization [36,37,38]. These techniques require hospitalization and premedication with intravenous hyperhydration over 24 to 48 h, antibio-prophylaxis (prevention of liver abscess), somatostatin analogs (to avoid carcinoid symptoms), antiemetics, and painkillers before and during hospitalization for 48 h after the procedure. Trans-arterial chemoembolization with streptozotocin must be performed under general anesthesia because the intrahepatic injection is painful. 

#### 1.4.2. Results

Results of trans-arterial embolization and trans-arterial chemoembolization are described in Table 1. The first study reporting on trans-arterial embolization treatment in NETs was published by Carrasco et al. in 1986 [39]. Many studies and reviews have since been published. Despite a large number of studies, the vast majority of trans-arterial embolization and trans-arterial chemoembolization studies are retrospective. Randomized placebo-controlled studies are lacking. Furthermore, a limited number of patients with various NETs (but the majority of small-bowel and pancreatic NETs) were included. Finally, the technique of trans-arterial embolization and trans-arterial chemoembolization is very heterogeneous. The efficacy of trans-arterial embolization and trans-arterial chemoembolization was quickly confirmed by the rapid efficacy on the secretory syndrome, particularly on carcinoid syndrome or insulinoma. Symptom regression has been reported in 42% to 100% of patients [39,40,41,42,43,44,45,46,47,48,49,50,51,52,53,54,55,56,57,58,59,60]. Imaging criteria for assessing tumor response were the RECIST evaluation in computed tomography scans, but this was not detailed in all publications. The morphological response rate [39,40,41,42,43,45,46,47,48,49,50,51,52,53,55,56,57,58,59,60,61,62] was heterogeneous: 8% to 94% (median: 49%) for complete and partial response and 10% to 56% (median: 27%) for stable disease. De-vascularization induced by trans-arterial embolization or trans-arterial chemoembolization was not described as a tumor response in these studies. The progression-free survival [39,40,42,44,46,47,48,49,50,51,52,53,56,57,58,59,60,61] and overall survival [39,40,42,43,44,45,46,48,50,51,53,54,55,58,59,60] after trans-arterial embolization/trans-arterial chemoembolization were about 5 to 36 (median: 18.5) months and 16 to 69 (median: 34.5) months, respectively. A comparison between trans-arterial embolization and trans-arterial chemoembolization is difficult because the majority of studies are retrospective, with few patients, and no differentiation between small-bowel and pancreatic tumors. There were no statistical differences between trans-arterial embolization and trans-arterial chemoembolization in terms of progression-free survival or overall survival. Tolerance was improved with trans-arterial embolization [57,59]. In a prospective study, Maire et al. analyzed trans-arterial embolization and trans-arterial chemoembolization in small-bowel NETs. The 2–year progression-free survival was similar in trans-arterial embolization and trans-arterial chemoembolization. Efficacy was not improved by the use of chemotherapy in comparison with embolization alone, which does not favor the addition of chemotherapy for these tumors. This study was closed before completion. Hepatic arterial embolization (embolization, chemoembolization, and radio-embolization) is recommended by ENETS and NANETS in patients with liver metastases who are not candidates for surgical resection [7,19,20].

#### 1.4.3. Complications

Trans-arterial embolization and trans-arterial chemoembolization are quite safe procedures. Del Prete et al. published a review including 896 NETs treated by trans-arterial embolization or trans-arterial chemoembolization [37]. Complications were observed in 14% and death occurred in 6% of cases. The most frequent adverse event was post-embolization syndrome [27,37]. It included abdominal pain, nausea, fever, hypertension, thrombocytopenia, leukocytosis, increase in transaminases, and lactate dehydrogenase. This occurs in up to 90% of patients [27]. Other severe complications include asthenia, liver necrosis with major cytolysis and/or liver failure, renal insufficiency, liver abscesses, ischemic gastritis (ulcers, bleeding), ischemic cholecystitis, and carcinoid crisis in small-bowel NETs with carcinoid syndrome. Furthermore, repeated trans-arterial embolization and chemoembolization can cause arteritis and liver artery stenosis. Complications are more frequent when the whole liver is treated, rather than targeting each segment separately [46]. 

### 1.5. Trans-Arterial Radio-Embolization 

#### 1.5.1. Techniques 

The rational for trans-arterial radio-embolization (TARE) is the same as trans-arterial embolization and trans-arterial chemoembolization. Radio-embolization or trans-arterial radio-embolization is a percutaneous trans-arterial injection of micron-sized embolic particles loaded with a radioisotope. In the case of LM, trans-arterial radio-embolization was performed with 30-µm-sized 90Yttrium microspheres of glass (Thera-Sphere) or resin (SIR-Spheres) and was administrated through a catheter directly into the hepatic arteries. 90Yttrium is a β-emitter and decays to stable 90Zirconium with a half-life of 64 h. Millions of radioactive microspheres (15 to 20 million if resin, 1 to 8 million if glass) are selectively released into the hepatic artery. This technique has been developed to target multiple sites; the number and sites of liver metastases do not limit its use [35]. This treatment preferentially delivers a high dose of radiation to the tumor while sparing the normal liver.

#### 1.5.2. Results 

Trans-arterial radio-embolization is an emerging treatment. This treatment is indicated in clinical practice is as a first-line therapy, as a second-or-more-line therapy or as salvage therapy for refractory disease. Results of trans-arterial radio-embolization are described in Table 2. Symptom regression has been reported in 55% to 100% (median: 89.5%) of patients [63,64,65,66,67,68]. The morphological response rate with RECIST evaluation in computed tomography scans was heterogeneous: 12% to 73% (median: 51%) for complete and partial response and 15% to 75% (median: 33%) for stable disease [63,64,65,66,67,68,69,70,71,72,73,74,75,76]. The progression-free survival and the overall survival after trans-arterial radio-embolization were about 9 to 11 months (median: 10 months) and 14 to 70 months (median: 28.5 months), respectively [63,64,65,68,69,70,71,72,73,74,75,76]. Radio-embolization is recommended by NANETS in patients with liver metastases who are not candidates for surgical resection [19,20]. ENETS guidelines highlight that more safety data on long-term tolerability are required [7]. 

#### 1.5.3. Complications 

Trans-arterial radio-embolization has a low toxicity profile [69]. The most common side effects of radio-embolization are abdominal pain, nausea, fever, and fatigue. Other complications resulting from non-targeted delivery of radio-embolization products are liver dysfunction, radiation gastritis and gastric or duodenal ulcer, and radio-induced pneumonia. For King et al., there was one early death from liver dysfunction among 34 patients [63]. 

### 1.6. Peptide Receptor Radionuclide Therapy 

#### 1.6.1. Techniques

The high-level expression of somatostatin receptors in the majority of NETs provides the basis for tumor-targeted therapy. Peptide receptor radionuclide therapy (PRRT) uses radiolabeled small peptides, such as somatostatin analogues (DOTATOC, DOTATATE, or DOTANOC), that target somatostatin receptors on NETs. Theses analogues are labelled with ß-emitting radionuclides, such as Lutetium-177 (177Lu) or Yttrium-90 (90Y). 177Lu-DOTATATE is currently the most widely used radiopeptide for PRRT [77]. The PRRT regimen consists of four administrations of a fixed dose of 177Lu-DOTATATE every 8 weeks. Each administration was given in conjunction with anti-nausea medication and an amino acid formulation to protect kidney function (lysine and arginine) [78]. 

#### 1.6.2. Results

The clinical efficacy of PRRT has been demonstrated in several retrospective studies [79,80,81,82,83,84,85], mainly for midgut NETs. These studies focused on the outcome and safety of PRRT. The response rate (response and stable disease) was about 74% to 100% (median: 86%) [79,80,81,82,83,85] for 177Lu-DOTATATE and 90Y-DOTATOC. The progression-free survival [79,80,81,82,83,84,85] and overall survival [79,80,81,82,85] were 16 to 36 (median: 29) and 22 to 55 (median: 37) months, respectively. A recent retrospective study with 44 patients analyzed the long-term outcome of PRRT after a 12-year follow-up. The median overall survival was 79 months. In total, 32% of patients with metastatic disease were still alive [86]. The Neuroendocrine Tumors Therapy (NETTER-1) trial was the first randomized controlled trial that evaluated the efficacy and safety of 177Lu-DOTATATE in patients with advanced, progressive, and somatostatin receptor-positive midgut NETs compared to the standard conventional treatment (long-acting octreotide). The response rate was 18% in the 177Lu-DOTATATE group, significantly higher than the 3% in the control group. The median progression-free survival was 28.4 months after PRRT, in comparison to 8.4 months in the control group (*p* < 0.0001). The overall survival for the PRRT arm has not yet been reached [87]. ENETS guidelines recommend the use of PRRT in midgut NETs with metastases as a second-line therapy after progression under somatostatin analogues [7]. To date, no phase 3 trial of PRRT has been conducted in patients with NETs with a pancreatic primary site, but a large retrospective study of 443 patients with NETs (all primary sites) confirmed the efficacy of PRRT in the overall patient group of bronchial and gastrointestinal and pancreatic NETs [88]. Currently, PRRT is not yet recommended by ENETS.

#### 1.6.3. Complications

The toxicity profile is favorable: PRRT was well tolerated with only a few serious side-effects. Acute side effects are transient nausea or vomiting, related to the administration of kidney-protective amino acids. The main subacute side effect is mild and reversible hematologic toxicity, occurring within 4 to 6 weeks after therapy. Long-term side effects of PRRT may include renal failure and acute leukemia or myelodysplastic syndrome. A recent review of toxicities post-PRRT in 2225 patients found that acute leukemia or myelodysplastic syndrome occurred in only 1.4% of patients [89]. 

## 2. Conclusions

No randomized studies have shown the superiority of one treatment over another. These locoregional therapies are not recommended for predominant extrahepatic metastatic diseases or neuroendocrine carcinoma. Indications typically include secretory syndrome resistant to conventional medical treatments, and/or tumor growth control. Due to patients’ general good condition and the “long course” of their disease (well-differentiated NETs), they may receive several successive loco-regional treatments alternating with periods of simple monitoring or medical treatment. The treatment objective is to maintain a “good” quality of life. The main criteria to choose a treatment over another was the histological grade, time to progression, percentage of liver invasion, LM localization, and significant side effects of each treatment. Furthermore, these therapies may be considered repetitively. In a patient with functional NETs, specific symptom-controlling measures can be performed before LM treatment (like carcinoid syndrome with somatostatin analogs to prevent carcinoid crisis). The European (ENETs) recommendations are useful in therapeutic decisions made, if possible, in multidisciplinary meetings [7].

Surgery with curative intent of the primitive and LM is recommended as a first-line treatment. This strategy has been demonstrated in a recent meta-analysis [90], with a significantly improved 5-year overall survival after LM resection vs. no resection at all, vs. chemotherapy and embolization. Selecting criteria are a well-differentiated tumor, absence of extrahepatic metastasis, and simple pattern of LM (unilobar or limited extension) [14]. This treatment can be performed in a one or two-step surgical strategy [91].

Local ablation techniques (percutaneously or laparoscopically) can be performed as a sole treatment or in combination with surgery in the case of well-differentiated NETs. The indication of radiofrequency ablation only is the presence of limited LM with surgery contraindicated. Radiofrequency ablation with surgery is indicated in a case of a complex pattern of LM (bilobar extension) to limited major liver resection. The metastatic lesion should measure less than 5 cm. Radiofrequency ablation may be contraindicated for lesions that are close to vital structures or the liver surface. 

In the case of diffuse LM, non-surgical treatments, like intra-arterial hepatic treatment, can be proposed. Ideally, this treatment is recommended for hypervascularized metastases in standard imaging, with a tumor less than 50% of the hepatic volume, after resection of the primary tumor (especially for pancreatic tumor). The indications for liver-directed treatments are: G1 or G2 NETs, with unresectable tumor progression and predominant hepatic metastasis, and a non-functional or poorly controlled secretory syndrome. The contra-indications are thrombosis of the portal vein, hepatocellular insufficiency, poor general status, predominant extrahepatic liver disease, and undifferentiated neuroendocrine carcinoma. Major hepatic invasion (>50%) requires a guided embolization. Bilio-digestive anastomosis and previous multiple radio-frequency destruction are relative contra-indications because they increase the risk of infection. Liver-directed treatments include TAE, TACE and TARE [36]. No studies comparing the three forms of liver therapies were found. In clinical practice, trans-arterial embolization and trans-arterial chemoembolization are used more frequently than trans-arterial radio-embolization. No statement on which arterial therapy offers the best progression-free survival and overall survival can be made [35]. Kalinowski et al. [72] suggest that the acute toxicity of trans-arterial radio-embolization is lower than trans-arterial embolization/trans-arterial chemoembolization. Best indications are not well recognized, but trans-arterial embolization and trans-arterial chemoembolization can be proposed in first-line or more treatment for small-bowel tumors, and in second- or third-line or more treatment as an alternative to systemic chemotherapy for pancreatic tumors. The place of trans-arterial radio-embolization is more limited [27,35,92]. In selected patients, the use of intra-arterial liver treatment can result in cytoreduction as a neoadjuvant treatment for potential surgery [93]. 

Debulking surgery is not indicated with a curative intent. It is, however, indicated in selected patients in the case of functional NETs with predominant liver disease to improve syndrome control, after the failure of medical treatment alone. The most frequent NETs treated by debulking surgery are NETs with carcinoid syndrome, refractory insulinoma, glucagonoma, or vasoactive intestinal peptide tumor (VIPoma) [14,15]. Indications for debulking or palliative resection are inadequate because of the morbidity and mortality of surgery [35]. Comparative trials to systemic treatment are lacking [14]. In the case of this surgery, it is important to prevent carcinoid crisis in NETs with carcinoid syndrome.

Finally, liver transplantation may be an option and should be discussed in precise selected cases with the following criteria: ki-67 < 10%, young age, absence of extrahepatic disease, resection of the primary tumor, hepatic invasion <50%, and tumor disease stable for at least 6 months [7,23].

Loco-regional treatments for LM of gastrointestinal NETs remain a major weapon. They combine good tolerance and frequently have a very long-lasting effectiveness. They should always be preferred before the introduction of a systemic treatment. In addition, loco-regional therapies propose a higher concentration of hepatic treatment compared to systemic treatment in cases of predominant hepatic invasion. However, they are reserved for expert centers with radiological, surgical, and oncological skills. These expert centers implement a multidisciplinary approach for personalized treatments, according to their specific experience of the specialized center while taking into account the current guidelines. Patients must be referred to these centers and therapeutic management should always be decided in multidisciplinary tumor meetings. 

In the future, these treatments could be included in randomized clinical trials, and combined with systemic treatments. As an example, the phase II study EVACEL (FFCD 1104) is performed to determine whether 24-month treatment with Everolimus prolongs progression-free survival rates (based on a central assessment) after embolization or chemoembolization of liver metastases. The current ongoing trials that address non-pharmacological treatments of NETs metastatic to the liver are shown in Table 3.

## Figures and Tables

**Figure 1:**
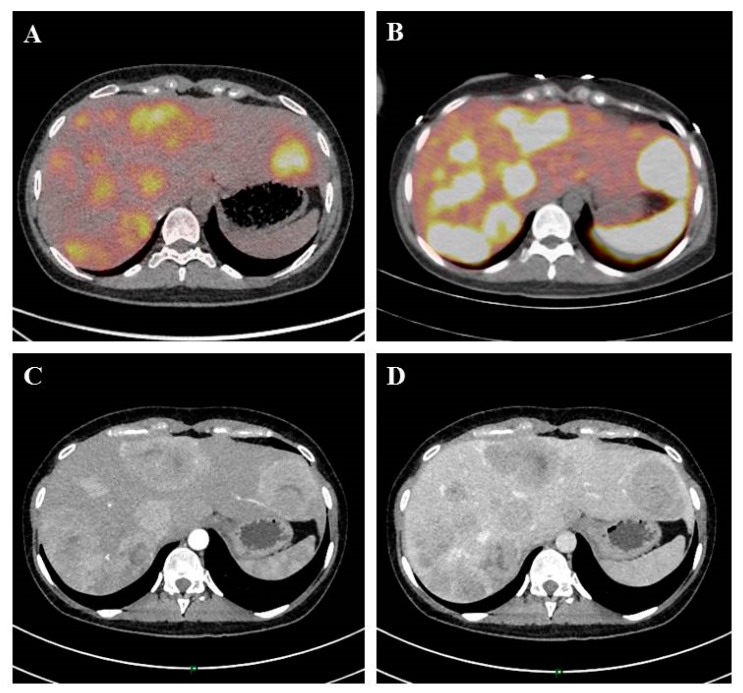
A 24-year-old patient with liver metastasis of a pancreatic neuroendocrine tumor. Initial evaluation identified liver metastases on ^18^ F-FDG PET Computed tomography scan (**A**), ^68^Ga-DOTATOC (**B**), computed tomography scan at the arterial phase (**C**), and computed tomography scan at the venous portal phase (**D**).

**Table 1:** Outcomes of studies of trans-arterial embolization and chemoembolization in patients with liver metastases from gastrointestinal Neuroendocrine tumors NETs.

Author, Years	*N*	Tumor Type			Treatment	Methods	Symptom Response (%)	Imaging Response		Survival	
		Small Bowel	Pancreas	Other				Tumor Response Rates (%) *	Stable Disease	Progression-Free Survival (Month)	Overall Survival (Month)
Carrasco, 1986	25	16	-	9	TAE	Sponge	87	94	-	11	16
Therasse, 1993	23	23	-	-	TACE	Doxo + sponge	100	35	24	29	24
Diamandidou, 1998	20	17	3	-	TACE	Cispl	67	78	22	-	-
Kim, 1999	30	16	14	-	TACE	Cisp + doxo	-	25	-	24	15
						5FU + STZ		50			
Yao, 2001	20	10	10	-	TACE	Doxo + mito + cispl	50	25	10	32	40
Kress, 2003	26	12	10	4	TACE	Doxo		8	53		
Loewe, 2003	23	23	-	-	TAE	Cyanoacrylate	56	73	23	-	69
Roche, 2003	14	14	-	-	TACE	Doxo + sponge	90	72	14		47
Osborne, 2006	59	42	17	-	TAE	PVA or embosphères	91			22	24
Strosberg, 2006	84	59	20	5	TAE	PVA or embosphères	80	48	52	-	36
Bloomston, 2007	122	122	-	-	TACE	Doxo + mito + cispl	92	82	12	19	33
Granberg, 2007	15	7	-	8	TAE	Embosphères	42	35	56	6	-
Ho, 2007	46	31	15	-	TAE or	Sponge or PVA	78	45	32	23^$^	42^$^
					TACE	Doxo + mito + cispl	75	45	45	16^$^	44^$^
Marrache, 2007	67	48	19	-	TACE	STZ or doxo	91	37	36	15	-
Varker, 2007	27	13	4	10	TACE	Doxo + mito + cispl	77	61	-	5	28
Christante, 2008	77	37	15	25	TAE or TACE	5FU	61	58	22	19	39
De Baere, 2008	20	20		-	TACE	DEB	-	80	15	15	-
Kamat, 2008	38	7	10	21	TAE or TACE	PVA or sponge Multiple chemotherapy	65	44	-	9	19
Pitt, 2008	100	56	44	-	TAE or TACE	Sponge, PVA, embosphere	TAE 76				26
						Cispl, adriamycin, mitomycin C	TACE 69				
Dong, 2011	123	21	61	41	TACE	Doxo, STZ	-	62	24	-	65$
Gaur, 2011	18	18	-	-	TACE	DEB doxo	-	58	42	14	
Maire, 2012	26	26	-	-	TAE or TACE	Sponge	-	65	30	24	
						Doxo				19	
Hur, 2013	46	22	-	-	TACE	Doxo	-	58	-	16	39
Fiore, 2014	30	16	12	2	TAE	Lipiodol	-	30	-	36	60
					TACE	Epirubicin		38	-		
Dhir, 2017	91	35	22	34	TACE	STZ	54	23	47	18	44

TAE: Trans-arterial embolization. TACE: Trans-arterial chemoembolization. Doxo: Doxorubicin. Cispl: Cisplatin. 5FU: 5-fluorouracil. STZ: streptozotocin. DEB: Drug-eluting beads trans-arterial chemoembolization. PVA: Polyvinyl alcohol. * Tumor response rates include complete and partial responses. ^$^ Mean.

**Table 2:** Outcomes of studies of trans-arterial radio-embolization in patients with liver metastases from gastrointestinal Neuroendocrine tumors (NETs).

Author, Years	*N*	Tumor Type			Treatment	Line	Symptom Response (%)	Imaging Response		Survival	
		Small Bowel	Pancreas	Other				Tumor Response Rates (%) *	Stable Disease (%)	Progression -Free Survival (Month)	Overall Survival (Month)
Kennedy, 2008	148	100	28	20	SIR-Spheres	1^st^	-	70	25	-	70
King, 2008	34	15	8	11	SIR-Spheres + 5FU infusion	1^st^ or more	55	50	15	-	24
Murthy, 2008	8	1	6	1	SIR-Spheres	Last	-	12	50	-	14
Rhee, 2008	42	31	11		SIR-Spheres	-	-	52	41	-	28
					Theraspheres						22
Kalinowski, 2009	9	4	3	2	SIR-Spheres	-	-	67	33	11	-
Cao, 2010	58	21	14	23	SIR-Spheres	-	-	39	27	-	36
Saxena, 2010	41	22	15	4	SIR-Spheres	1^st^	-	54	23	-	35
Lacin, 2011	13	3	3	7	SIR-Spheres	2^nd^ or more	-	50	40	-	18
Rajekar, 2011	14			14	SIR-Spheres SIR-Spheres + 5FU infusion	-	100	100		-	25
Ezzidin, 2012	23		14	9	SIR-Spheres Therasheres	Last	80	30	61	-	29
Memon, 2012	40	10	9	21	Theraspheres	1^st^	84	64	-	-	34
Paprottka, 2012	42	23	9	10	SIR-Spheres	Last	95	22.5	75	-	-
Ozao-Choy, 2013	18	14	3	1	SIR-Spheres	-	-	58	32	-	-
Peker, 2015	30	6	7	17	SIR-Spheres	2^nd^ or more	-	46	67	-	39
Fidelman, 2016	11	6	3	2	TheraSpheres	Last	100	73	27	9	-

5FU: 5-fluorouracil. * Tumor response rates include complete and partial responses. SIR: Selective internal radiation.

**Table 3:** Ongoing clinical trials about non-pharmacological treatment of NETs metastatic to the liver.

Study Title	Identifier	Type of Study	Recruiting Status	Sponsor
Selective Intra-arterial Injection of PRRT in Neuroendocrine Tumor Patients with Liver Metastases	NCT03724409	Early Phase 1	Recruiting	Sandeep Laroia
Randomized Embolization Trial for NeuroEndocrine Tumor Metastases to The Liver	NCT02724540	Phase 2	Recruiting	University of Pennsylvania
Pembrolizumab and Liver-Directed Therapy in Well-Differentiated Neuroendocrine Tumors with Liver Metastases	NCT03457948	Phase 2	Recruiting	Nicholas Fidelman, MD
Neo-adjuvant Peptide Receptor Mediated Radiotherapy With 177 Lutetium in Front of Curative Intended Liver Transplantation in Patients with Hepatic Metastasis of Neuroendocrine Tumors (NEO-LEBE)	NCT01201096	Observational	Unknown	University of Jena
Phase II Study of Sunitinib Malate Following Hepatic Artery Embolization	NCT00434109	Phase 2	Completed	H. Lee Moffitt Cancer Center and Research Institute
DEBOXA for Inoperable NET Liver Metastases	NCT03881306	Phase 1	Recruiting	Xiangya Hospital of Central South University
		Phase 2		
Stereotactic Body Radiation Therapy (SBRT) for Unresectable Liver Metastases	NCT02185443	Phase 2	Recruiting	University of Sao Paulo
Everolimus After (Chemo)Embolization for Liver Metastases from Digestive Endocrine Tumors (EVACEL)	NCT01678664	Phase 2	Active, not recruiting	Federation Francophone de Cancerologie Digestive
Efficacy and Safety of 177Lu-edotreotide PRRT in GEP-NET Patients (COMPETE)	NCT03049189	Phase 3	Recruiting	ITM Solucin GmbH
Antitumor Efficacy of Peptide Receptor Radionuclide Therapy With 177Lutetium -Octreotate Randomized vs Sunitinib in Unresectable Progressive Well-Differentiated Neuroendocrine Pancreatic Tumor: First Randomized Phase II (OCCLURANDOM)	NCT02230176	Phase 2	Recruiting	Gustave Roussy, Cancer Campus, Grand Paris
